# Very Broadly Effective Hemagglutinin-Directed Influenza Vaccines with Anti-Herpetic Activity

**DOI:** 10.3390/vaccines12050537

**Published:** 2024-05-14

**Authors:** David C. Bloom, Cameron Lilly, William Canty, Nuria Vilaboa, Richard Voellmy

**Affiliations:** 1 Department of Molecular Genetics & Microbiology, University of Florida College of Medicine, Gainesville, FL 32610-0266, USA; dbloom@ufl.edu (D.C.B.); clilly@ufl.edu (C.L.); williamcanty@ufl.edu (W.C.); 2 Hospital Universitario La Paz-IdiPAZ, 28046 Madrid, Spain; nuria.vilaboa@salud.madrid.org; 3 CIBER de Bioingenieria, Biomateriales y Nanomedicina, CIBER de Bioingenieria, Biomateriales y Nanomedicina, 28046 Madrid, Spain; 4 HSF Pharmaceuticals SA, 1814 La Tour-de-Peilz, Switzerland

**Keywords:** vaccine, vectored vaccine, influenza, broad protection, replication-competent, conditionally replicating, regulated, universal flu vaccine

## Abstract

A universal vaccine that generally prevents influenza virus infection and/or illness remains elusive. We have been exploring a novel approach to vaccination involving replication-competent controlled herpesviruses (RCCVs) that can be deliberately activated to replicate efficiently but only transiently in an administration site in the skin of a subject. The RCCVs are derived from a virulent wild-type herpesvirus strain that has been engineered to contain a heat shock promoter-based gene switch that controls the expression of, typically, two replication-essential viral genes. Additional safety against inadvertent replication is provided by an appropriate secondary mechanism. Our first-generation RCCVs can be activated at the administration site by a mild local heat treatment in the presence of an antiprogestin. Here, we report that epidermal vaccination with such RCCVs expressing a hemagglutinin or neuraminidase of an H1N1 influenza virus strain protected mice against lethal challenges by H1N1 virus strains representing 75 years of evolution. Moreover, immunization with an RCCV expressing a subtype H1 hemagglutinin afforded full protection against a lethal challenge by an H3N2 influenza strain, and an RCCV expressing a subtype H3 hemagglutinin protected against a lethal challenge by an H1N1 strain. Vaccinated animals continued to gain weight normally after the challenge. Protective effects were even observed in a lethal influenza B virus challenge. The RCCV-based vaccines induced robust titers of in-group, cross-group and even cross-type neutralizing antibodies. Passive immunization suggested that observed vaccine effects were at least partially antibody-mediated. In summary, RCCVs expressing a hemagglutinin induce robust and very broad cross-protective immunity against influenza.

## 1. Introduction

Vaccination has been highly successful in the prevention of many infectious illnesses. However, important infections have remained refractory to effective vaccination. Respiratory illness caused by type A and type B influenza viruses is a prime example of an illness that is not adequately prevented by vaccination, in an important part because the viruses evolve relatively rapidly by antigenic drift and antigenic shift. Seasonal influenza has been estimated to cause between 290,000 and 650,000 deaths worldwide each year (ref. [1]; https://www.who.int/en/news-room/fact-sheets/detail/influenza-(seasonal), accessed on 5 April 2024). The envelopes of influenza viruses contain two major glycoproteins, hemagglutinin (HA) and neuraminidase (NA). Data suggest that the majority of antibodies induced by natural infection are directed against HA, and only a minority of antibodies are directed against NA and other proteins [2]. The HA protein comprises an immunodominant, variable globular head domain and a subdominant, more conserved stem (or stalk) domain. Strain-specific neutralizing antibody responses induced by natural infection or conventional vaccination are directed largely to the variable head domain. Influenza A viruses are subdivided into subtypes and strains based on similarities between HA and NA proteins, respectively. The 18 HA subtypes are assigned to two distinct phylogenetic groups. Group 1 comprises subtypes H1, H2, H5, H6, H8, H9, H11, H12, H13, H16, H17 and H18, and group 2 subtypes H3, H4, H7, H10, H14 and H15. Influenza B virus strains are separated into the B/Yamagata/16/88-like and the B/Victoria/2/87-like lineages. Typical seasonal influenza vaccines are subunit vaccines that comprise HAs from contemporaneously circulating H1N1 and H3N2 influenza A strains and at least one influenza B strain (https://iris.who.int/bitstream/handle/10665/336951/9789240010154-eng.pdf, accessed on 5 April 2024). Live attenuated influenza virus vaccines have also been licensed. These vaccines essentially only protect against homologous virus strains. In addition, their protective effects wane rapidly, in contrast with the longer-lasting immunity induced by natural infection [2,3,4]. The continuing evolution of the influenza viruses and the relatively narrow protective effects of conventional vaccination prompted the establishment of the WHO’s Global Influenza Surveillance and Response System (GISRS), which attempts to predict the most appropriate vaccine strains for the next influenza season (https://www.who.int/initiatives/global-influenza-surveillance-and-response-system, accessed on 5 April 2024). The U.S. CDC estimated overall vaccine effectiveness through the U.S. VE Network for most influenza seasons from 2004/2005 to 2023/2024 (https://www.cdc.gov/flu/vaccines-work/past-seasons-estimates.html, accessed on 12 May 2024). Vaccines were maximally effective in the 2010/2011 season (60%) and minimally effective in the 2004/2005 season (10%). Particularly low levels of effectiveness may be explained primarily by mismatch between vaccine strains and actually circulating strains, but infection/vaccination history may also play a role. Significant protection against pandemic outbreaks cannot be expected from seasonal influenza vaccines.

It has long been recognized that a new generation of influenza vaccines needs to be developed that are broadly cross-protective and induce long-lasting immunity (reviewed in refs. [2,3,4,5]; https://ivr.cidrap.umn.edu/, accessed on 5 April 2024). Various approaches for broadening the immune response to HA-directed vaccines have been explored in mice and other animal models. Priming with a DNA construct expressing an H1 HA and boosting with an H1 HA-containing vaccine elicited stem-directed neutralizing antibodies and conferred protection against diverse H1N1 strains [6]. In another approach, in which stem-directed HA antibodies were induced, the vaccine consisted of nucleoside-modified, purified mRNA encoding a full-length H1 HA formulated in lipid nanoparticles [7]. Vaccination protected mice against heterologous (H1N1) and heterosubtypic (H5N1) lethal challenges. Ferritin nanoparticles displaying rationally designed H1 HA induced stem- and receptor-binding domain-directed antibodies that neutralized various H1N1 strains and protected ferrets against an unmatched H1N1 strain [8]. It is noted that the above studies described in-group protective effects. No cross-group protection or protection against influenza B viruses was reported. A particularly promising strategy has been to deliberately enhance the immune response to the HA stem domain. This has been achieved either by immunization with headless HA proteins or mini-proteins or by sequential immunization with chimeric HA proteins that contained a common stem domain and head domains from different strains or subtypes [9,10,11,12,13,14,15,16,17,18,19]. Immunization with headless HA proteins or mini-proteins (based on or modeled after H1 or H5 HAs) protected mice and, in some studies, ferrets against heterologous and heterosubtypic lethal challenges [9,10,11,12,13,14]. In some but not all of the studies, immunization also effectively protected against illness. Several of the studies demonstrated by passive immunization that the protective effects of the stem immunogens were antibody-mediated. Cross-group protection was generally not observed, although partial cross-protection from lethality but not from illness was reported [13,14]. Sequential vaccination with chimeric HAs that contained a common stem domain from a subtype H1 HA protected animals essentially completely from lethality and illness upon challenge by heterologous (H1N1) or heterosubtypic group 1 viruses (H5N1, H6N1), but not by a group 2 virus (H3N2) [15]. When the chimeras contained a common stem region from a group 2 HA or a type B HA, immunization provided an effective defense against group 2 or type B viruses, respectively [16,17]. Inactivated subtype H3 viruses in which the major antigenic sites in the HA head domain had been replaced with sequences from an avian subtype H14 HA (mosaic HAs) afforded better protection against heterologous H3N2 viruses than a typical seasonal vaccine [20]. In another study, the immunogen was a subtype H1 HA stem region-CD40 ligand fusion protein [21]. Cross-group protection from lethality was observed in the immunized mice. However, all animals, including animals challenged with an H1N1 influenza virus strain, became ill, as evidenced by severe weight loss. Another strategy involved the generation of computationally optimized, broadly reactive antigenic (COBRA) HAs that induced a broadened antibody response against heterologous viruses [22,23,24]. Many of the latter approaches have been advanced to the clinic [5]. Also entering a first clinical trial is a vaccine that consists of inactivated avian influenza viruses of HA subtypes H1, H3, H5 and H7 [25]. The vaccine protected mice against lethal challenges with viruses of subtypes present in the vaccine as well as against certain subtypes (H6, H10) not present in the vaccine. However, the vaccine failed to prevent illness in mice challenged with viruses of the latter subtypes, as evidenced by weight loss. A 20-valent nucleoside-modified mRNA candidate vaccine encoding HAs from all influenza A virus subtypes and influenza B virus lineages was recently described [26]. The vaccine protected mice against lethal challenges with antigenically matched and mismatched H1N1 viruses, although significant weight loss occurred in the animals challenged with the mismatched virus. Neutralizing antibodies were induced against the antigenically matched but not the mismatched virus. Finally, a caspase-attenuated live intranasal influenza vaccine protected mice against cross-group lethal challenges, although weight loss was observed with some of the challenge viruses [27]. Notably, the vaccine failed to elicit neutralizing antibodies, and antibody-mediated cellular cytotoxicity was low. Hence, what mediated the protective effects remains unknown, and the safety of the self-attenuation mechanism, especially in immunocompromised subjects, has yet to be demonstrated.

Herpes simplex viruses of type 1 and 2 (HSV-1 and HSV-2) cause genital herpes, and HSV-1 infection is a major cause of blindness. HSV infection is associated with significant morbidity and even mortality. Recent estimates have the worldwide prevalence of HSV-1 at 67% (people under the age of 50) and that of HSV-2 at 13% (people aged 15–49) (https://www.who.int/news-room/fact-sheets/detail/herpes-simplex-virus, accessed on 5 April 2024; see also refs. [28,29]). An effective preventative vaccine against HSV-1 and/or HSV-2 remains elusive [30,31]. Research efforts continue, and several novel vaccine candidates have shown efficacy in animal models [32,33,34,35,36,37]. 

We have been pursuing the hypothesis that a virus vector that replicates efficiently but in a temporally and spatially controlled fashion (referred to herein as “replication-competent controlled virus”, abbreviated as “RCCV”) induces a more potent and more complete immune response than an attenuated vector or a replication-defective vector (and, presumably, a subunit vaccine). First-generation RCCVs were constructed using a virulent HSV-1 strain as the backbone [38,39]. In these recombinants, one or two replication-essential viral genes are subjected to the control of a dual-responsive gene switch that can be activated transiently. Using a stringent mouse footpad lethal challenge model, we found that vaccination with RCCVs that were activated locally in the administration region protected mice against a lethal HSV-1 challenge far more effectively than a replication-defective comparison HSV-1 strain or not-activated RCCVs [39]. HSV-1-specific antibody and responder cell responses correlated well with the protective responses. 

Here, we report on the functional immune responses elicited by RCCVs expressing influenza virus antigens in mouse models.

## 2. Materials and Methods

### 2.1. Cells and Viruses

Rabbit skin (RS) cells were a gift of E. Wagner, Vero cells were procured from the American Type Culture Collection (ATCC, Manassas, VA, USA), and Vero-derived E5 cells [40] were provided by N. DeLuca. RS cells were cultured in minimal essential medium Eagle’s salts (MEM) (Life Technologies, Thermo Fisher Scientific, Waltham, MA, USA) supplemented with 5% heat-inactivated calf serum (Atlanta Biologicals, Lawrenceville, GA, USA), 292 μg/mL L-glutamine, 250 U/mL of penicillin and 250 µg/mL of streptomycin (Life Technologies). Vero and E5 cells were cultured in Dulbecco’s modified Eagle’s medium (DMEM) supplemented with 10% heat-inactivated fetal calf serum, 250 U/mL of penicillin and 250 µg/mL of streptomycin. E5 cells were used to propagate stocks of HSV-GS3, HSV-GS19, HSV-GS21, HSV-GS25, HSV-GS26 and HSV-GS27. Infected cultures were incubated in medium supplemented with 10 nM ulipristal and subjected to heat treatment at 43.5 °C for 30 min for 3 consecutive days. Stocks were tittered as described under “Single-step growth analysis”. Influenza virus strains A/California/07/2009(H1N1), A/Puerto Rico/8/1934(H1N1), A/Fort Monmouth/1/1947(H1N1), A/Solomon Islands/3/2006(H1N1) and B/Brisbane/60/2008 were obtained from BEI Resources (Manassas, VA, USA), and strain A/Hong Kong/4801/2014(H3N2; mouse-adapted) was a gift of E. B. Tarbet. The influenza virus strains were propagated on Madin-Darby canine kidney (MDCK) cells (ATCC) cultured in DMEM supplemented with 10% heat-inactivated horse serum, as previously described [41]. MDCK cells were used to titer viral stocks using 50% tissue culture infectious dose (TCID_50_) assays. All cells were cultured at 37 °C under 5% CO_2_.

### 2.2. Chemical Reagents

Ulipristal acetate (USP grade) was procured from D-Innovation Pharmaceutical Inc., Chengdu, Sichuan, China.

### 2.3. Generation of RCCVs

Wild-type HSV-1 strain 17*syn*+ was used as the backbone to construct all RCCVs. To generate viral recombinants by homologous recombination, RS cells were co-transfected with engineered plasmids along with purified virion DNA by the calcium phosphate precipitation method [42]. The construction of HSV-GS3 was described previously [38]. HSV-GS19 was derived from recombinant HSV-GS3 and contains a gene cassette, inserted between the UL37 and UL38 genes, expressing the HA gene of strain A/California/07/2009 driven by the CMV IE promoter. As also reported previously [39], a recombination plasmid was constructed using the following sequential steps. First, an 814 bp fragment from plasmid NK470, including the region that spans the UL37/UL38 intergenic region of HSV-1 strain 17syn+ (nt 83,603–84,417), was inserted into pBluescript (pBS) that had the multiple-cloning site removed by digestion with KpnI/SacI to yield pBS:UL37/38 [39]. To prepare the plasmid pIN:UL37/38, a cassette containing a synthetic CMV IE promoter flanked by the pBS-SK+ multiple-cloning site was inserted into pBS:UL37/38 digested with BspE1/AflII, enzymes that cut between the UL37 and UL38 genes. A codon-optimized version of the full-length HA gene of A/California/07/2009 (Genbank accession KU933485) was synthesized by GeneScript (Piscataway, NJ, USA) and subcloned into pBS. The HA gene was subsequently excised from the latter plasmid and inserted behind the CMV promoter in the plasmid pIN:UL37/38 to yield plasmid pIN:37/38-Cal/07/HA. This plasmid was co-transfected along with purified HSV-GS3 virion DNA in RS cells to produce recombinant HSV-GS19. The co-transfected cells were exposed to ulipristal and then heated by partially immersing the sealed dishes in a 43.5 °C water bath for 30 min, followed by incubation at 37 °C. Subsequently, on days 2 and 3, the cultures were again incubated at 43.5 °C for 30 min and then returned to 37 °C. Plaques were isolated and amplified in E5 cells cultured on 96-well plates in medium supplemented with ulipristal. One h after infection, the cultures were incubated at 43.5 °C for 30 min and then further incubated at 37 °C. Subsequently, on days 2 and 3, the plates were again subjected to the same heat treatment and then returned to 37 °C. After a 90–100% cytopathic effect was observed, the plates were dot-blotted, and the dot-blot membrane hybridized with a DNA probe prepared by ^32^P-labeling the synthetic HA gene. Several positive plaques were identified, and two were subjected to 3 rounds of plaque purification. Initial stocks were prepared for Southern blot analysis and sequencing of the insert. One recombinant clone was verified by Southern blot analysis as well as by sequencing of the HA insert and ~200 bp of the flanking sequence on each side of the insert. Upon this genetic verification, a master stock was prepared. The ability of this stock to produce the HA protein was confirmed by enzyme-linked immunosorbent assay (ELISA) on lysates of cells infected at a multiplicity of infection (MOI) of 3 and heat-treated in the presence of ulipristal. The latter lysates were prepared at 12 h post-infection. Recombinants HSV-GS21, HSV-GS25, HSV-GS26 and HSV-GS27 were constructed analogously. Synthetic, full-length genes (codon-optimized) for the NA of A/California/07/2009 (Genbank accession NC_026434) (HSV-GS21), the HAs of A/Hong Kong/4801/2014 (GISAID EpiFlu 653201) (HSV-GS26) and A/Perth/16/2009 (Genbank accession KJ609206) (HSV-GS27) as well as the HA stem fragment immunogen gene H1HA10-FOLDON [13] (HSV-GS25) were procured from GeneScript. Sequences of the cloned inserts were verified by Sanger sequencing. Expression of NA from HSV-GS21 and of HAs from HSV-GS26 and HSV-GS27 was verified by ELISA. RNA transcripts of the H1HA10-FOLDON gene expressed from HSV-GS25 were detected by RT-PCR at 12 h post-infection. Detection of the protein product using an H1N1 polyclonal serum was not successful. 

### 2.4. Single-Step Growth Analysis

An RCCV was added to confluent monolayers of Vero cells at a MOI of 3. Following adsorption at 37 °C for 1 h, the inoculum was removed, and the cells were incubated in complete medium. Ulipristal exposure (10 nM) was initiated during RCCV adsorption. Immediately after infection, heat treatment was performed at 43.5 °C for 30 min followed by incubation at 37 °C. At 0, 4, 12 and 24 h post-infection, the cells from 3 dishes were scraped into the medium for harvesting, and lysates were prepared by two freeze-thaw cycles. To determine infectious virus, the lysate of each dish was tittered in triplicate on 24 well plates of confluent E5 cells transfected 24 h prior to infection with pICP8 [38], an ICP8 expression vector, using Lipofectamine 2000 (Life Technologies). Two days after infection, plaques were visualized using an antibody plaque assay, essentially as described previously [38].

### 2.5. Immunization

An RCCV or phosphate-buffered saline (PBS; pH 7.3) (50 µL/mouse) was applied to the lightly abraded plantar surfaces of both rear feet of 4- to 6-week-old female BALB/c, DBA/2 or JHT mice (Envigo, Tampa, FL, USA), as previously described [42]. The feet were saline-treated prior to infection to minimize the amount of abrasion required and to facilitate efficient uptake of the virus. Mice were anesthetized with isoflurane by inhalation. Then, flunixin meglumine (1.1 mg/kg) was administered intramuscularly (IM) to alleviate any suffering associated with the procedure. Thereafter, 25–50 µL (no more than 50 µL) of sterile 10% saline (10% NaCl *w*/*v*) were administered subcutaneously under both rear footpads. The mice were then returned to their cages. Four h later, the mice were anesthetized by IM administration of 10–20 µL of a cocktail of ketamine (30–45 mg/kg), xylazine (7.5–11.5 mg/kg) and acepromazine (2.5–3.75 mg/kg). The keratinized layer of the skin of both rear footpads was scratched by light abrasion with an emery board to allow for efficient virus adsorption. The mice were placed on their backs, and 50 µL of the appropriate dilution of the RCCV or PBS was administered on the footpads using a pipette. The viral recombinant was allowed to adsorb until the mice awoke. A combination of heat and ulipristal treatment was used to activate RCCV replication. Three h after virus administration, heat treatment was performed by immersion of hindlimbs for 10 min in a temperature-controlled water bath at 44.5 °C. Then, mice were allowed to recover at 37 °C for 15 min. Ulipristal (50 µg/kg), dissolved in dimethylsulfoxide (DMSO), was administered by intraperitoneal (IP) injection at the time of virus inoculation and routinely again 24 h later. It is noted that we did not explore whether this second application of ulipristal enhanced vaccine performance. Typically, mice were immunized twice, i.e., the above procedure was repeated 3 wk after the initial immunization.

### 2.6. Challenge Experiments

In experiments relating to protection against influenza, immunized and control mice were challenged by intranasal administration of, depending on the strain, 1 × 10^1^ to 5 × 10^5^ TCID_50_ doses of an influenza virus strain. Challenged animals were monitored daily (with cages coded in a masked fashion), and their weights were recorded. A weight loss of more than 20% was used as the clinical endpoint in accordance with the approved protocol. Animals that reached the clinical endpoint were euthanized. For HSV-1 challenges, immunized and control mice were inoculated on both rear footpads with 1 × 10^4^ PFU/mouse, typically of HSV-1 strain 17syn+. Saline pre-treatment, anesthesia and application of the virus were performed as described under “Immunization”. A modified endpoint analysis was used for the efficacy determination. Mice were housed in cages coded in a masked fashion and were monitored daily. Mice were euthanized when they reached clinical endpoints that indicated severe CNS infection, such as bilateral hindlimb paralysis, inability to move when touched or trembling.

The conclusions reached from animal experiments were based on results obtained from at least two independent experiments and redundancies within individual experiments (unless indicated otherwise).

### 2.7. Passive Immunization

Aliquots of 1 mL of undiluted or 10-fold diluted serum from RCCV-immunized BALB/c mice were administered IP to naïve adult BALB/c mice. Twenty-four h later, the animals were challenged intranasally with a lethal dose of either influenza virus strain A/California/07/2009 or A/Hong Kong/4801/2014. Animals were observed daily for 21 days, and weights were recorded.

### 2.8. Virus Replication in Mice

After infection of the footpads, 3 or 4 mice from each group were euthanized, and the rear feet were dissected at 48 h post-inoculation. The feet were snap-frozen in liquid nitrogen, pulverized with a sterile pestle in a sterile mortar and resuspended in 200 µL of lysis buffer (100 mM NaCl, 10 mM Tris [pH 8.0], 0.5% sodium dodecyl sulfate [SDS] and 0.1 mg of proteinase K per mL). Samples were then incubated for 3 h at 55 °C, extracted once with phenol-chloroform (1:1) and once with chloroform-isoamyl alcohol (24:1), ethanol precipitated and resuspended in Tris-ethylenediaminetetraacetic acid (EDTA) (10 mM Tris, 1 mM EDTA, pH 8.0). The purified DNA was then subjected to qPCR (StepOnePlus Real-Time PCR System; Applied Biosystems, Thermo Fisher Scientific, Waltham, MA, USA) with TaqMan Fast Universal PCR Master Mix (2X) (Applied Biosystems) and custom primers/probe-specific for the HSV-1 DNA polymerase gene (UL30). Limits of detection and relative quantities of HSV-1 DNA were determined by spiking in known numbers of copies of plasmid DNA containing the UL30 target region into DNA prepared from the feet of uninfected mice, as described above. 

### 2.9. Lung Titers of Challenge Virus

Mice were euthanized by isoflurane overdose at the indicated time points. Lungs were removed by dissection, weighed and then flash-frozen in liquid nitrogen and stored at −80 °C. To assay for infectious virus, frozen lungs were pulverized with a sterile pestle and ground in a glass homogenizer containing 1 mL of DMEM. The contents were transferred to a 2 mL Eppendorf tube, and the homogenizer was rinsed with 0.5 mL of medium, which was transferred to the tube. The tube was centrifuged at 2000× *g* at 4 °C to clarify the homogenate, and the supernatant was removed and transferred to a fresh tube. Ten-fold serial dilutions of the homogenate were used to infect 24 well dishes of confluent MDCK cells and assayed for TCID_50_.

### 2.10. Blood Collection

For serum neutralization analyses, blood was collected prior to immunization, prior to second immunization as well as prior to challenge. Mice were IP anesthetized with ketamine (90 mg/kg) and xylazine (10 mg/kg), and blood was collected by retro-orbital bleeding. Serum was collected at 3 wk after (second) immunization. Mice were anesthetized by inhalation of 2–3% isoflurane, and the total blood volume of each mouse was withdrawn by cardiac puncture. Then, the mice were euthanized by an overdose of isoflurane anesthesia followed by cervical dislocation.

### 2.11. Microneutralization Assay

Collected blood was allowed to clot for 30 min and then centrifuged at 800× *g* to separate the serum from blood cells. To inactivate complement, serum samples were heated to 56 °C for 1 h, and then were diluted 1:10 in complete DMEM supplemented with 10% heat-inactivated fetal bovine serum. MDCK cells were seeded into 96-well plates at 3 × 10^4^ cells/well and incubated overnight at 37 °C under 5% CO_2_. Eight serial, two-fold dilutions were prepared from serum samples in infection medium (DMEM w/o serum), with a starting dilution of 1:16. An appropriate influenza virus, diluted to yield 0.1 TCID_50_/well, was combined with an equal volume of diluted serum sample and then incubated for 1 h at 37 °C. After incubation, the medium from the pre-seeded cells was replaced with the virus/serum mixtures. After incubation for 12–18 h at 37 °C under 5% CO_2_, cells were fixed with 3.7% formaldehyde in PBS for a minimum of 10 min. Following fixative removal, cells were permeabilized by incubation in PBS containing 0.1% Triton-X-100. Cells were stained for 1 h with 50 μL/well of a cocktail consisting of the detection antibodies for the specific influenza strain, each diluted to 500 ng/mL. Cells were washed with 0.1% Triton-X-100 in PBS and then incubated for 1 h with 50 μL/well of a solution of horseradish peroxidase (HRP) secondary antibody, diluted 1:400 in PBS containing 0.05% Tween-20 (PBST) and 3% non-fat dried milk (blocking solution). After washing with 200 μL/well of PBST, HRP substrate was added to the plates and allowed to develop. Then, the plates were washed with PBS, dried and read using a plate reader to determine the neutralization endpoints.

### 2.12. HA ELISA

ELISA plates were coated overnight with 50 μL/well of dilutions of the test samples in PBS with protease inhibitors. After washing with PBST and, subsequently, with blocking solution, the plates were incubated for 1 h with 50 μL/well of 10 µg/mL rabbit anti-HA antibody. Plates were washed with PBST and then incubated for 1 h with 100 μL/well of goat anti-rabbit secondary antibody (Invitrogen 31460, Thermo Fisher Scientific) diluted 1:2000 in PBST. Plates were washed with PBST and incubated at room temperature for 4 min with 100 μL/well of SuperBlu-Turbo TMB substrate (Virolabs, Chantilly, VA, USA). Following the addition of 100 μL/well of Stop solution (Virolabs), plates were read at OD 450 nm.

### 2.13. Statistical Analyses

Unless otherwise indicated in the figure legends, data are presented as mean values with standard deviation. The Statistical Program for Social Sciences version 25 (IBM Corp.; Armonk, NY, USA) was employed to analyze the data. Kaplan–Meier survival curves were assessed for significance using the log-rank test. To evaluate whether the data followed a normal distribution, Kolmogorov–Smirnov normality tests were conducted. One-way analysis of variance (ANOVA) followed by Bonferroni’s multiple comparison test was conducted to analyze the parametric data from 3 or more groups, while nonparametric data were analyzed using the Kruskal–Wallis test followed by Mann–Whitney U-test for post hoc group comparisons. The Student’s *t*-test and Mann–Whitney U-test were conducted to compare two groups with normally and non-normally distributed data, respectively. The sample size of the datasets that were assessed for statistical significance did not vary by more than 3-fold. For all comparisons, the criterion for significance was set at 0.05.

## 3. Results

### 3.1. RCCVs Expressing an Influenza Virus HA

**Figure 1:**
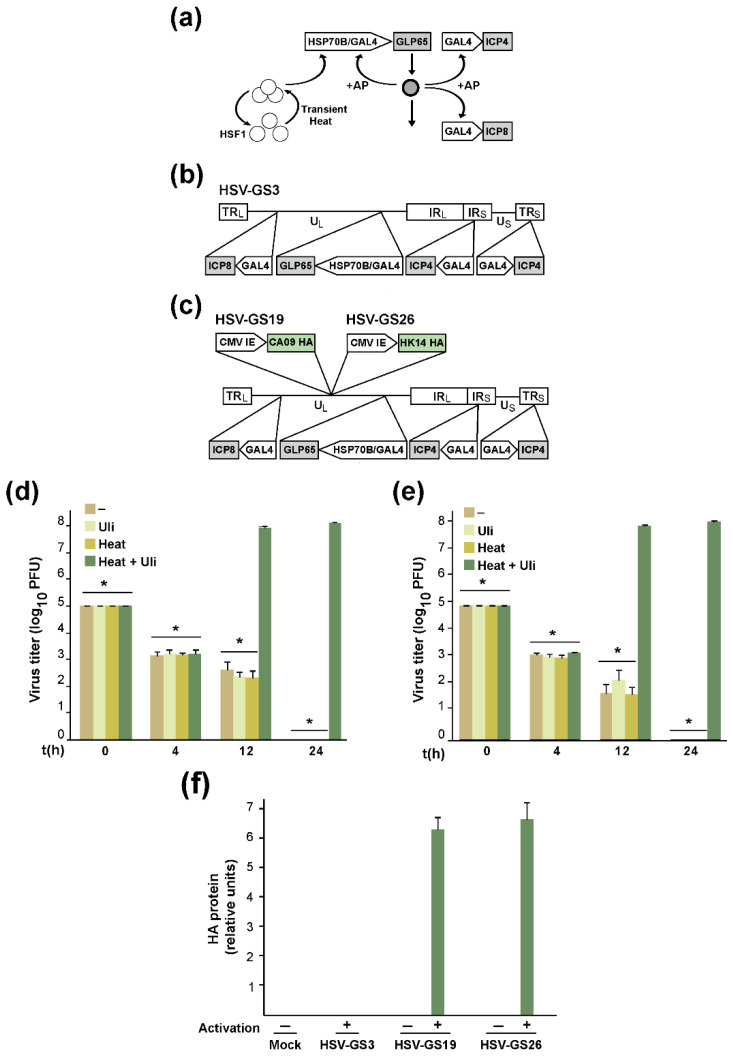
Two-component gene switch, schematic representation of the structures of RCCVs, regulation of RCCV replication and expression of HAs from RCCVs. (**a**) Dually responsive gene switch in HSV-GS recombinants: a promoter assembly comprising an HSP70B promoter (HSP70B) and a GAL4-responsive promoter (GAL4) controls a gene for antiprogestin (AP)-activated transactivator GLP65. The replication-essential ICP4 and ICP8 genes are controlled by GAL4 promoters. Heat treatment of a cell infected with an HSV-GS recombinant transiently activates the cellular heat shock factor (HSF1) that then transactivates the GLP65 gene. Newly synthesized, inactive GLP65 molecules are activated when bound by an AP. Activated GLP65 transactivates the GAL4 promoter-controlled ICP4 and ICP8 genes as well as its own gene. (**b**) Diagram of RCCV HSV-GS3. The recombinant comprises, inserted in the UL43/44 intergenic region of HSV-1 wild-type strain 17syn+, transactivator gene GLP65, which is functionally linked to an HSP70B/GAL4 (GAL4-responsive promoter) promoter assembly. GLP65 is a chimeric transcription factor comprising a yeast-derived GAL4 DNA-binding domain, an antiprogestin-binding domain derived from the ligand-binding domain of a human progesterone receptor and an activation domain from the human P65 protein. Antiprogestin-activated GLP65 transactivates GAL4-responsive promoters. The native promoters of the replication-essential genes encoding ICP4 (both copies) and ICP8 in HSV-1 strain 17syn+ were replaced with GAL4-responsive promoters. (**c**) Diagram of RCCVs HSV-GS19 and HSV-GS26. The RCCVs are derived from HSV-GS3 and additionally comprise a CMV IE promoter-driven gene encoding the HA of influenza virus strain A/California/07/2009 (CA09) (HSV-GS19) or A/Hong Kong/4801/2014 (HK14) (HSV-GS26) inserted in the UL37/38 intergenic region. TR_L_, TR_S_: long and short terminal repeats; U_L_, U_S_: long and short unique regions; IR_L_, IR_S_: long and short internal repeats. (**d**,**e**) Gene switch-controlled replication of RCCVs HSV-GS19 and HSV-GS26. Single-step growth experiments with HSV-GS19 (**d**) and HSV-GS26 (**e**) were carried out in Vero cells. Heat: cultures were exposed to 43.5 °C for 30 min immediately after infection (i.e., immediately after removal of the viral inoculum); Uli: 10 nM ulipristal was added to the medium at the time of infection. Mean values of the results of 3 individual assays are presented. The values are expressed as log_10_ total plaque-forming units (PFU). * *p* ≤ 0.05 (compared with cells treated with heat and ulipristal at 12 h or 24 h). (**f**) Regulated expression of HAs from RCCVs HSV-GS19 and HSV-GS26. Adult BALB/c mice (groups of 3 mice) were inoculated on their rear footpads either with saline (mock) or with HSV-GS3, HSV-GS19 or HSV-GS26 (all at 5 × 10^4^ PFU). Activation was by a 10 min immersion of the hindlegs in a 45 °C water bath 3 h after inoculation in the presence of ulipristal (50 µg/kg; administered IP at the time of virus administration). Tissue samples were harvested from mouse feet 24 h later, and protein homogenates were prepared and analyzed by HA-specific ELISA. The data represent the mean values of the chromogenic signals of the samples minus the mean value of the negative control relative to the mean value of the negative control.

First-generation RCCV, HSV-GS3, was derived from virulent HSV-1 strain 17syn+ [38]. In this recombinant, the replication-essential genes for infected cell proteins 4 and 8 (ICP4 and ICP8) are controlled by a gene switch that is armed by an antiprogestin (AP) and can be activated transiently by subjecting an infected host cell to a heat treatment. The gene switch comprises a gene for transactivator GLP65. GLP65 combines a GAL4 DNA-binding domain, a truncated ligand-binding domain from a human progesterone receptor, and a human P65 activation domain. The transactivator is expressed under the control of a promoter assembly consisting of a human HSP70B (HSPA7) promoter and a GAL4-responsive promoter. The GLP65 gene cassette was inserted into the UL43/UL44 intergenic region of wild-type HSV-1 strain 17syn+, and the native promoters of the ICP4 and ICP8 genes were replaced with GAL4-responsive promoters [38]. The operation of the gene switch is illustrated in Figure 1a (see also ref. [43]), and the structure of HSV-GS3 is shown schematically in Figure 1b. RCCV HSV-GS3 replicated efficiently in different mammalian cell lines and in vivo in mice after heat treatment to the infected cells in the presence of AP mifepristone or ulipristal [38]. No significant replication was detected in the absence of this activation treatment or in the absence of either heat treatment or AP. It is noted that a heat-activated mechanism might have been adequate for controlling the replication of an RCCV vaccine in an inoculation site. The AP co-control was included for additional safety, in particular, to prevent inadvertent systemic replication of the RCCV vaccine or replication/reactivation in nerve cells under adverse conditions (e.g., a high fever). 

RCCV HSV-GS19 was derived from HSV-GS3 by the insertion into the UL37/UL38 intergenic region of a cytomegalovirus (CMV) promoter-controlled, full-length gene for the HA of the pandemic influenza virus strain A/California/07/2009(H1N1) (CA09). RCCV HSV-GS26 was constructed analogously and comprised an inserted full-length HA gene of H3N2 strain A/Hong Kong/4801/2014(H3N2) (HK14). The structures of HSV-GS19 and HSV-GS26 are depicted schematically in Figure 1c. Single-step growth experiments demonstrated that the recombinants were unable to detectably replicate in untreated cells, in cells subjected to a heat treatment or in cells exposed to ulipristal (Figure 1d,e). They replicated efficiently after the infected cells were heat-treated in the presence of ulipristal (activation treatment). Robust HA expression was detected in mouse feet one day after inoculation with activated RCCV HSV-GS19 or activated HSV-GS26 (Figure 1f). HA levels in animals that received activated recombinant HSV-GS3 or not-activated recombinants HSV-GS19 or HSV-GS26 were not significantly above the assay background. It is noted that because their HA genes are driven by unregulated promoters, not-activated RCCVs HSV-GS19 and HSV-GS26 are capable of HA expression. That HA levels in infected feet were low is explained by the absence of replication. For easy reference, the RCCVs employed in the present study and the influenza virus antigens they express are listed in Table 1.

### 3.2. Protection against a Lethal Challenge by a Homologous Influenza Virus Strain

**Figure 2:**
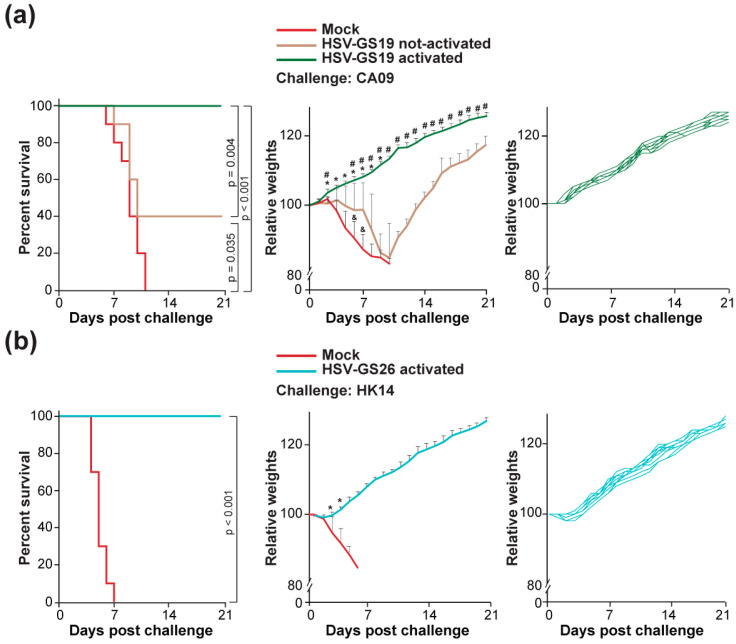
Homologous influenza virus challenges. Groups (n = 10) of adult BALB/c mice were inoculated on the slightly abraded plantar surfaces of their rear feet with 2.5 × 10^5^ PFU of RCCV HSV-GS19 or HSV-GS26 or vehicle. RCCV replication was activated by a local heat treatment in the systemic presence of ulipristal. Heat treatment for 10 min at 44.5 °C was performed by immersion of hindlimbs in a temperature-controlled water bath 3 h after virus administration. Ulipristal (50 µg/kg) in DMSO was administered IP at the time of virus inoculation. Inoculations (with the same RCCVs and at the same doses of RCCVs) and activation treatments were repeated 3 wk later. After a further 3 wk, all mice were challenged intranasally with a lethal dose of the homologous influenza virus strain. Animals were observed daily, and weights were recorded. Left graphs: survival (≤20% weight loss) after challenge; center graphs: averaged relative weights of surviving animals after challenge. Weights are relative to weights on the day of challenge. Relative values and standard deviations are shown. *p* ≤ 0.05 (compared with mock-immunized animals (*) or to animals immunized with not-activated HSV-GS19 (#, &)); right graphs: relative weights after challenge of all animals in the groups vaccinated with activated RCCV. Weights are relative to weights on the day of challenge. (**a**) Mice immunized twice with activated or not-activated RCCV HSV-GS19 (expressing the HA of influenza virus strain A/California/07/2009 (CA09)) or vehicle (mock) and challenged with a lethal dose of influenza virus strain CA09. (**b**) Mice immunized twice with activated RCCV HSV-GS26 (expressing the HA of influenza virus strain A/Hong Kong/4801/2014 (HK14)) or vehicle (mock) and challenged with a lethal dose of influenza virus strain HK14.

RCCVs HSV-GS19 or HSV-GS26 (10 animals per group) or vehicle were administered to the slightly abraded footpads (i.e., epidermally) of the hindfeet of adult BALB/c mice (2.5 × 10^5^ plaque-forming units (PFU) of RCCV per animal). RCCVs were activated by IP injection of ulipristal (50 µg/kg body weight) and localized heat treatment of the hindfeet (44.5 °C for 10 min). Three wk later, all mice were re-immunized and, after a further 3 wk, were challenged intranasally with a lethal dose of the homologous influenza virus strain, i.e., strain CA09 in the case of HSV-GS19-immunized animals and strain HK14 (mouse-adapted) in the case of HSV-GS26-immunized animals. All mice were observed daily for 3 wk, and survival and weights were recorded. Mice immunized with activated HSV-GS19 or HSV-GS26 were fully protected against the respective homologous lethal challenge (Figure 2a,b, left graphs). Average animal weights increased normally subsequent to the challenge (Figure 2a,b, center graphs). It is noted that young adult (10–12 wk-old) BALB/c mice are well-known to continue gaining weight (see, e.g., ref. [34], Figure 2A). None of the animals showed any weight loss or signs of distress (Figure 2a,b, right graphs). Not-activated HSV-GS19 afforded only poor protection against the homologous lethal challenge (Figure 2a). A majority of the mice succumbed to the challenge, and all surviving animals exhibited transient weight loss at some time during the observation period. That vaccination with activated recombinant HSV-GS3 affords no protection was confirmed in preliminary experiments.

Replication of the recombinants in vivo was stringently controlled by the gene switch. In an example experiment, two groups of three mice were inoculated with HSV-GS19. Activation treatment was administered to one of the groups. Two days later, the animals were sacrificed, and DNA was extracted from their hind feet. Quantitative PCR analysis using primers/probe specific for HSV-1 DNA polymerase detected 2300 ± 450 viral genomes/mg tissue in the animals that were subjected to activation treatment but failed to detect viral genomes in the animals that had not received an activation treatment (detection limit: 0.15 genomes/mg tissue). 

### 3.3. Protection against Heterologous Lethal Challenges

**Figure 3:**
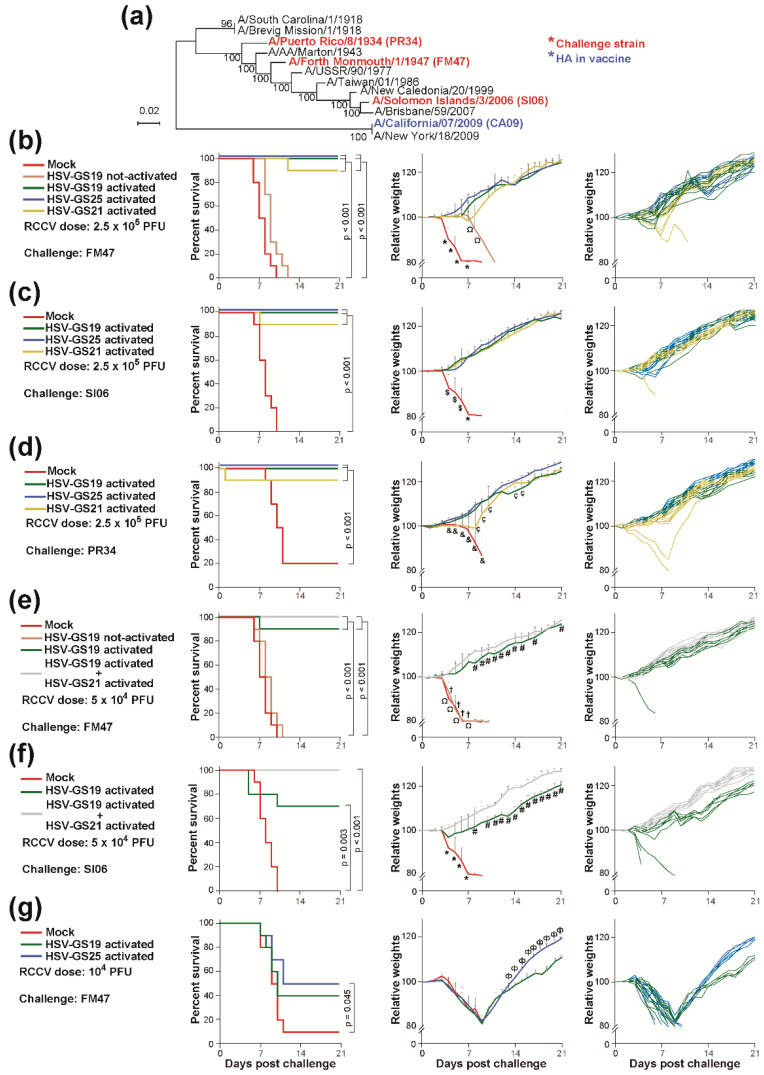
Heterologous (intra-subtypic) influenza virus challenges. (**a**) Diagram illustrating the phylogenetic relationships between the HA expressed by RCCV-GS19 and the HAs of the challenge virus strains employed (adapted from ref. [44]). (**b**–**d**) Groups (n = 10) of adult BALB/c mice (or DBA/2 mice for challenges with strain A/Solomon Islands/3/2006 (SI06)) were immunized twice with 2.5 × 10^5^ PFU/mouse of not-activated or activated RCCV HSV-GS19, activated recombinants HSV-GS21 or HSV-GS25, or vehicle as detailed in Figure 2. RCCV HSV-GS19 expresses a full-length HA of H1N1 strain A/California/07/2009 (CA09), RCCV HSV-GS21, a full-length NA of strain CA09, and RCCV HSV-GS25, a stem region fragment containing HA sequences of strain A/Puerto Rico/8/1934 (PR34). Three wk after the second immunization, the mice were challenged intranasally with lethal doses of H1N1 influenza virus strains A/Fort Monmouth/1/1947 (FM47) (**b**), SI06 (**c**) or PR34 (**d**). The animals were observed daily, and their weights were recorded. Left graphs: survival (≤20% weight loss) after challenge; center graphs: averaged relative weights of surviving animals after challenge. Weights are relative to weights on the day of challenge. Relative values (down to the nadir in the case of control groups comprising surviving animals) and standard deviations are shown; right graphs: relative weights after challenge of all animals in the groups vaccinated with an activated RCCV. Weights are relative to weights on the day of challenge. (**e**,**f**) Similar experiment in which groups (n = 10) of adult BALB/c (**e**) or DBA/2 (**f**) mice were immunized twice with 5 × 10^4^ PFU/mouse of not-activated or activated HSV-GS19 or a combination of activated HSV-GS19 and HSV-GS21 (5 × 10^4^ PFU each), or vehicle and challenged with lethal doses of H1N1 strains FM47 (**e**) or SI06 (**f**). (**g**) Groups (n = 10) of adult mice were immunized twice with 1 × 10^4^ PFU/mouse of activated RCCVs HSV-GS19 or HSV-GS25, or vehicle and were challenged with strain FM47. Center graphs: *p* ≤ 0.05 (comparing the mock-immunized group to any RCCV-immunized group shown in the graph (*), to the activated HSV-GS19 or the HSV-GS21 group ($), to the activated HSV-GS19 or the HSV-GS25 group (&), or to the activated HSV-GS19 or the HSV-GS19/HSV-GS21 group (†), comparing the not-activated HSV-GS19 group with the activated HSV-GS19 or the HSV-GS19/HSV-GS21 group (Ω), comparing the activated HSV-GS19 group with the HSV-GS19/HSV-GS21 group (#) or the activated HSV-GS25 group (Φ) or comparing the activated HSV-GS21 group with the activated HSV-GS19 group (Ç)).

To find out whether vaccination with RCCV HSV-GS19 expressing the HA of H1N1 strain CA09 generated cross-protective immunity, groups (n = 10) of adult mice (BALB/c, or DBA/2 for challenge studies involving strain A/Solomon Islands/3/2006 (SI06)) were immunized twice with 2.5 × 10^5^ PFU/mouse of the RCCV or were mock-immunized as described above. The mice of most but not all immunized groups were subjected to an activation treatment. Three wk after the last immunization, all animals were inoculated intranasally with a lethal dose of a heterologous influenza virus strain and were then observed daily, and weights were recorded. As a comparison, parallel groups of mice were immunized with RCCV HSV-GS25, an HSV-GS3-derived recombinant expressing H1HA10-Foldon A and HA stem fragment immunogen containing sequences from the HA of A/Puerto Rico/8/1934 (PR34) [13]. The challenge viruses employed were H1N1 strains PR34, A/Fort Monmouth/1/1947 (FM47) and SI06. Based on phylogenetic relationships (Figure 3a; adapted from ref. [44], these strains appear to be reasonably representative of the evolution of H1N1 viruses over the 75-year time span ending with the pandemic of 2009. Hemagglutination inhibition assays reveal that sera from ferrets immunized with strains FM47, SI06 or A/New York/18/2009 are unable to neutralize heterologous virus strains from the group consisting of strains PR34, FM47, SI06 and A/New York/18/2009 (closely related to CA09) [44]. That the study failed to detect cross-reactivity attests to the relatively distant relationships of the latter strains. We found that vaccination with activated RCCV HSV-GS19 expressing the HA of strain CA09 completely protects against lethal challenge by the heterologous strains PR34, FM47 and SI06 (Figure 3b–d, left graphs). Average mouse weights increased steadily after the challenge (Figure 3b–d, center graphs). At the level of individual mice, none of the animals exhibited transient weight loss or any signs of distress (Figure 3b–d, right graphs). As shown for the FM47 challenge, essentially, no protection was afforded by the not-activated RCCV HSV-GS19. 

We also explored whether RCCV-based vaccination directed against an influenza virus NA could elicit comparable cross-protective anti-influenza immune responses. Groups of mice (n = 10) were immunized as described above with 2.5 × 10^5^ PFU/animal of RCCV HSV-GS21 (activated) or were mock-immunized. The latter recombinant was derived from HSV-GS3 by the insertion of an expressible, full-length NA gene from H1N1 strain CA09. Strong cross-protective effects were observed, although this protection appeared slightly less robust than that provided by vaccination with HA-expressing RCCV HSV-GS19 (Figure 3b–d, left graphs). No significant weight loss of averaged mouse weight was registered (Figure 3b–d, center graphs). Minor transient weight loss occurred in a minority of surviving mice (Figure 3b–d, right graphs).

Wondering whether protective effects afforded by vaccination against HA could be strengthened by co-vaccination against NA, groups of mice (n = 10) were immunized with a reduced dose (5 × 10^4^ PFU/animal) of activated HSV-GS19 alone or in combination with activated HSV-GS21 (at 5 × 10^4^ PFU/animal). Immunized and mock-immunized animals were challenged with lethal doses of strains FM47 or SI06. Lowering the administered dose appeared to reduce the vaccine efficacity of HSV-GS19, where the effect was more evident (*p* = 0.067) for the SI06 challenge (Figure 3e,f, left graphs). Co-vaccination with HSV-GS21 resulted in full protection against the heterologous strains. No loss of average weight nor in the weights of individual mice was observed (Figure 3e,f, center and right graphs). The mice continued to gain weight normally after the challenge.

Being unable to predict at the outset how efficacious HSV-GS19 vaccination would be against heterologous (intra-subtypic) challenges, HSV-GS25 was included as a potential positive control vaccine in the experiments reported in Figure 3b–d. RCCV HSV-GS25 directs the immune response to the conserved H1 HA stem region. Somewhat unexpectedly, HSV-GS19 and HSV-GS25 are found to be similarly effective. To detect potential differences in cross-protective efficacy, we immunized groups of mice (n = 10) with very low doses (1 × 10^4^ PFU/animal) of activated HSV-GS19 or HSV-GS25 and challenged the mice with a lethal dose of strain FM47. HSV-GS19 and HSV-GS25 had comparable partial protective effects (Figure 3g). Thus, in the RCCV context, a full-length HA elicits a similarly efficacious immune response against heterologous H1N1 influenza virus strains as a stem fragment antigen. Therefore, these results suggest that there is no reason to limit the HA antigens to their stem regions. 

**Figure 4:**
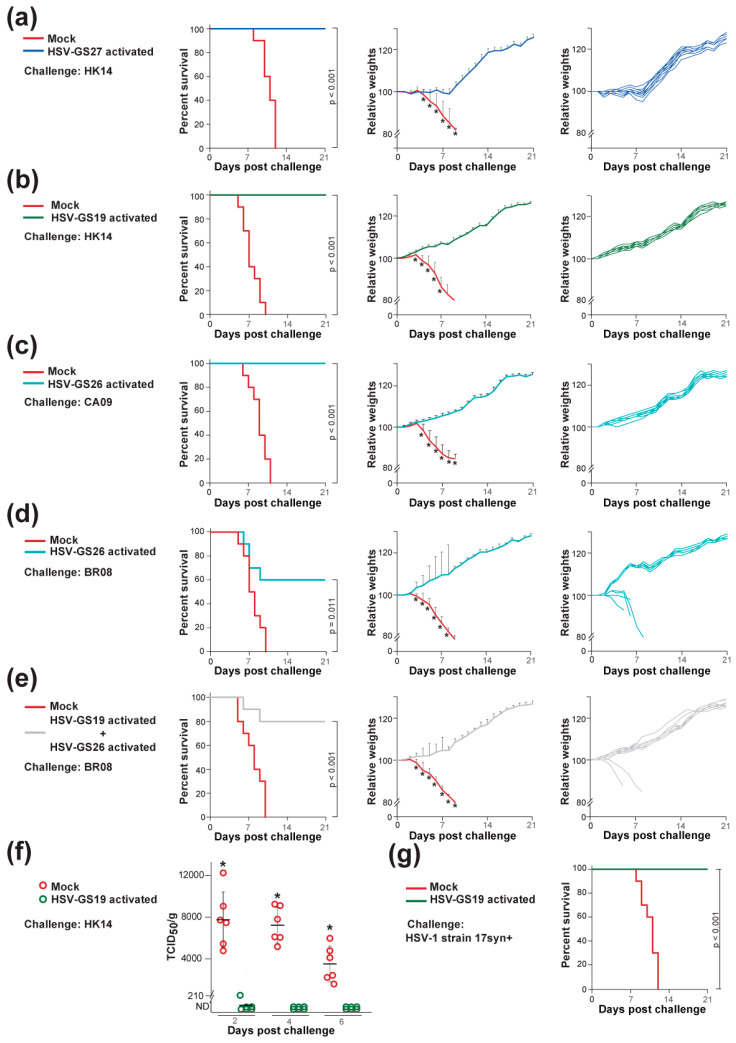
Heterologous (intra-subtypic), cross-group and cross-type influenza virus challenges, and an HSV-1 challenge. Groups (n = 10 or 30 in (**f**)) of adult BALB/c mice were immunized twice with 2.5 × 10^5^ PFU/mouse of the indicated activated RCCVs or vehicle and were challenged with a lethal dose of the indicated influenza virus strains as described in Figure 2. (**a**) Mice immunized with RCCV HSV-GS27 (expressing the HA of H3N2 influenza virus strain A/Perth/16/2009) or vehicle (mock) and challenged with H3N2 influenza virus strain A/Hong Kong/4801/2014 (HK14). (**b**) Mice immunized with RCCV HSV-GS19 (expressing the HA of H1N1 influenza virus strain A/California/07/2009 (CA09)) or vehicle (mock) and challenged with H3N2 influenza virus strain A/Hong Kong/4801/2014 (HK14). (**c**) Mice immunized with RCCV HSV-GS26 (expressing the HA of H3N2 influenza virus strain A/Hong Kong/4801/2014 (HK14)) or vehicle (mock) and challenged with H1N1 influenza virus strain A/California/07/2009 (CA09). (**d**) Mice immunized with RCCV HSV-GS26 (expressing the HA of H3N2 influenza virus strain A/Hong Kong/4801/2014 (HK14)) or vehicle (mock) and challenged with influenza B virus strain B/Brisbane/60/2008 (BR08). (**e**) As (**d**), except that animals were immunized with a combination of HSV-GS19 and HSV-GS26 (both at 2.5 × 10^5^ PFU/mouse). Left graphs: survival (≤20% weight loss) after challenge; center graphs: averaged relative weights of surviving animals after challenge. Weights are relative to weights on the day of challenge. Relative values and standard deviations are shown. * *p* ≤ 0.05 (compared with groups immunized with the indicated RCCV); right graphs: relative weights after challenge of all animals in the RCCV-vaccinated groups. Weights are relative to weights on the day of challenge. (**f**) Lung titers of challenge virus HK14. The results are presented as TCID_50_ values per g of tissue. Limit of detection: 210 TCID_50_/g tissue. ND: not detected. * *p* ≤ 0.05 (compared with groups immunized with RCCV). The experiment is described in the results. (**g**) HSV-1 challenge. A group (n = 10) of adult BALB/c mice that had previously been immunized twice with 2.5 × 10^5^ PFU/mouse of RCCV HSV-GS19 and challenged with a lethal dose of influenza virus strain FM47 and an age-matched control group were challenged (3 wk after the earlier challenge) with a lethal dose of HSV-1 wild-type strain 17syn+. Animals were observed daily for 3 wk, and survival was recorded.

Finally, we wished to learn whether or not RCCV-mediated protection against heterologous influenza viruses was limited to strains of subtype H1. Groups of mice (n = 10) that were immunized with activated HSV-GS27 or were mock-immunized were challenged with a lethal dose of H3N2 strain HK14 (clade 3C.2a). HSV-GS27 expresses the full-length HA gene of H3N2 strain A/Perth/16/2009 (clade 1). Animals vaccinated with activated HSV-GS27 were fully protected against the lethal heterologous challenge (Figure 4a, left graph). There was no significant loss in average weight subsequent to the challenge, although normal weight gain appeared delayed by several days (Figure 4a, center graph). This was also apparent at the level of individual mice (Figure 4a, right graph).

### 3.4. Protection against Cross-Group and Type B Virus Challenges

To test the limits of the RCCV-based vaccine approach, we asked whether an RCCV expressing an influenza type A HA could induce an immune response that is protective across influenza virus groups or even across types. Adult BALB/c mice (n = 10) were immunized as described above with activated RCCV HSV-GS19 (2.5 × 10^5^ PFU/animal) expressing an H1 HA (group 1) or activated recombinant HSV-GS26 (2.5 × 10^5^ PFU/animal) expressing an H3 HA (group 2). H1 HA-immunized animals were completely protected against a lethal challenge by H3N2 strain HK14 (Figure 4b, left graph). Conversely, H3 HA-immunized mice were fully protected against a lethal challenge by H1N1 strain CA09 (Figure 4c, left graph). No signs of distress or weight loss were observed after the challenges (Figure 4b,c, center and right graphs). Normal weight gain continued after the challenges. Significant partial protection (60%) against a lethal challenge by strain B/Brisbane/60/2008 (BR08; B/Victoria/2/87-like lineage) was afforded by vaccination with activated RCCV HSV-GS26 (Figure 4d, left graph) and, to a slightly lesser degree, by vaccination with activated recombinant HSV-GS19. No overall weight loss was observed (Figure 4d, center graph). It is noted that, while four animals rapidly succumbed to the challenge, the remaining six animals continued to gain weight normally, i.e., appeared to be completely protected (Figure 4d, right graph). Vaccination with a four-fold higher dose of HSV-GS26 only marginally improved survival. However, increasing the antigenic breath of the type A HA by vaccinating with both HSV-GS19 and HSV-GS26 (each at 2.5 × 10^5^ PFU/animal) resulted in 80% protection against the influenza B virus challenge (Figure 4e). The surviving eight animals gained weight normally after the challenge.

To assess viral replication in the lungs of vaccinated animals after the challenge, one group of adult BALB/c mice (n = 30) was immunized twice with activated recombinant HSV-GS19 (2.5 × 10^5^ PFU/animal), and another group was mock-immunized. At 2, 4 and 6 days after intranasal challenge with a lethal dose of influenza virus strain HK14, six animals of each of the groups were euthanized, lungs were recovered and virus extracted from lung tissues was tittered on MDCK cells as described in Section 2. Whereas elevated titers of the challenge virus were determined in mock-immunized animals at all time points (Figure 4f), the results revealed that essentially no replication of the virus occurred in the lungs of the vaccinated animals. Ten animals from each group were observed daily for 3 wk and survival was recorded. All animals of the vaccinated group but none of the mock-immunized group survived the challenge. Clinical endpoints were reached by the mock-immunized animals between 5 and 10 days after the challenge.

### 3.5. Protective Response against an HSV-1 Challenge

A group of adult BALB/c mice (n = 10) that had been twice immunized with 2.5 × 10^5^ PFU/animal of HSV-GS19 and challenged with influenza virus strain FM47, and age-matched not-immunized animals (n = 10) were subjected to a challenge by virulent HSV-1 strain 17syn+. Lethal doses of strain 17syn+ were administered epidermally to the rear footpads of all animals. The challenged animals were then observed daily for a period of 3 wk. All animals in the HSV-GS19 group fully recovered, whereas all animals in the control group reached the clinical endpoint (Figure 4g). These results demonstrate that vaccination with an HA does not blunt the immune response directed against HSV-1 antigens.

### 3.6. Serological Analyses

**Figure 5:**
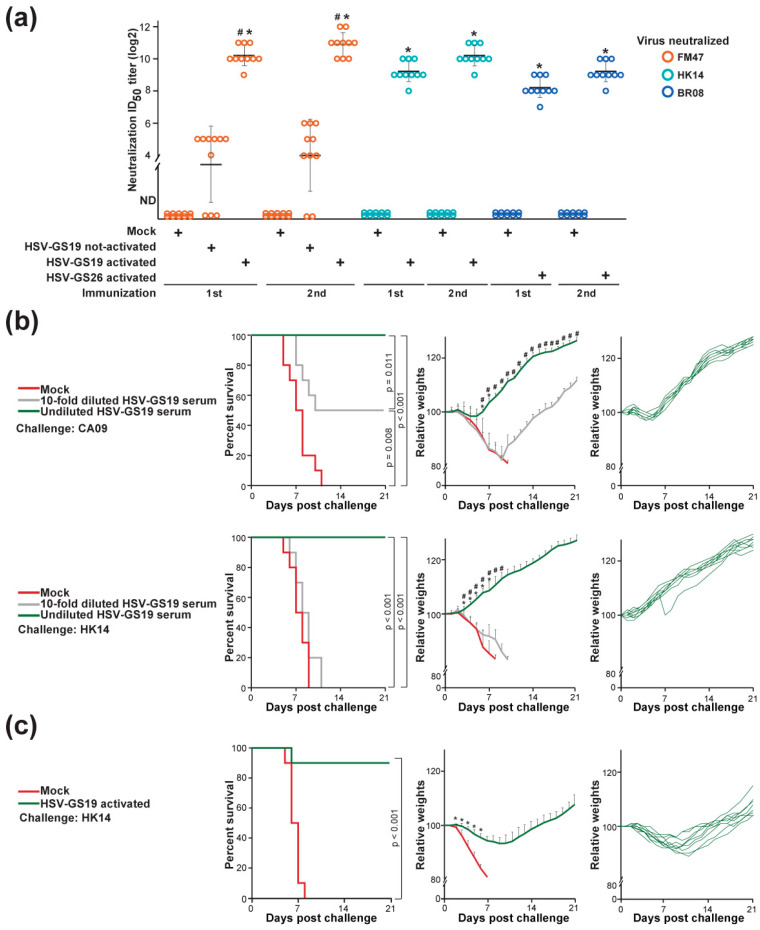
Neutralizing antibodies, passive immunization and a challenge experiment with JHT mice. (**a**) Neutralizing antibody responses. Groups of adult BALB/c mice (n = 10) were pre-bled and bled again 3 wk after the first immunization with the indicated RCCV or vehicle (mock) and 3 wk after the second immunization. Sera were prepared for each animal in the respective groups and analyzed for neutralizing antibodies against the indicated influenza virus strains using the microneutralization assay described in Section 2. Data are presented as neutralization ID_50_ titers (reciprocal dilutions where infection was reduced by 50% relative to normal serum expressed as geometric mean ID_50_). Lines indicate mean ± standard deviation. ND: not detected (below the limit of detection). *p* ≤ 0.05 (compared with the mock group (*) or the not-activated HSV-GS19 group (#)). (**b**) Passive immunization. Forty adult BALB/c mice were immunized twice with 2.5 × 10^5^ PFU/mouse of RCCV HSV-GS19. Three wk after the second immunization, total blood volumes were collected and combined and serum was prepared. Undiluted or 10-fold diluted serum (1 mL) was administered IP to groups of naive adult BALB/c mice (n = 10). Twenty-four h later, all animals were challenged with a lethal dose of either influenza virus strain A/California/07/2009 (CA09) or A/Hong Kong/4801/2014 (HK14). Left graphs: survival (≤20% weight loss) after challenge; center graphs: averaged relative weights of surviving animals after challenge. Weights are relative to weights on the day of challenge. Relative values and standard deviations are shown. *p* ≤ 0.05 (compared with the mock group (*) or to the group of animals administered 10-fold diluted serum (#)); right graphs: relative weights after challenge of all animals of the group that had received undiluted serum. Weights are relative to weights on the day of challenge. (**c**) Challenge experiment employing JHT mice. Groups (n = 10) of adult JHT mice were immunized twice with 2.5 × 10^5^ PFU/mouse of activated RCCV HSV-GS19 or vehicle and were challenged with a lethal dose of influenza virus strain HK14, as described in Figure 2. * *p* ≤ 0.05 (compared with the mock group). See under (**b**) for details of presentation.

Mice (10 animals per group) were pre-bled and then bled 3 wk after the first immunization and, again, 3 wk after the second immunization. Endpoint titers in sera were determined by a standard microneutralization assay. Neutralizing antibodies were not detected in sera from pre-bleeds. Sera obtained after a first immunization with activated RCCV HSV-GS19 expressing the H1 HA of strain CA09 (2.5 × 10^5^ PFU/animal) exhibited elevated titers of antibodies neutralizing heterologous H1N1 strain FM47 (Figure 5a). Titers of antibodies neutralizing H3N2 strain HK14 were only about two-fold lower (the difference not reaching statistical significance). Consistent with the lack of protective effects, antibody titers in sera from mock-immunized mice or from mice immunized with not-activated HSV-GS19 (2.5 × 10^5^ PFU/mouse) were not detectable or exceedingly low, respectively. Sera from mice immunized with activated RCCV HSV-GS26 (2.5 × 10^5^ PFU/mouse) expressing the H3 HA of strain HK14 were found to contain antibodies neutralizing type B strain BR08 at substantial titers. Neutralizing antibody titers appeared to be somewhat higher in sera obtained after the second immunization. 

### 3.7. Passive Immunization

Serum was prepared from adult BALB/c mice (n = 40) immunized twice with 2.5 × 10^5^ PFU/animal of activated RCCV HSV-GS19 (H1 HA). One mL/animal of undiluted or 10-fold diluted serum, or vehicle, was administered IP to groups of naïve adult BALB/c mice (n = 10). The mice were challenged 24 h later with a lethal dose of influenza virus strain CA09 (H1N1) or HK14 (H3N2). Mice that had received undiluted serum were protected completely, and those that had received diluted serum were protected partially against the lethal CA09 challenge (Figure 5b, top left graph). Mice injected with undiluted serum but not those injected with diluted serum were also protected against the lethal HK14 challenge (Figure 5b, bottom left graph). Mice passively immunized with undiluted serum experienced no significant weight loss after the challenge (Figure 5b, center and right graphs). These results support the notion that the protective effects of RCCV vaccines are at least in part antibody-mediated. 

### 3.8. T-Cell Response

To begin exploring whether vaccination with an RCCV expressing an influenza virus HA also induces a broadly protective HA-directed T-cell response, a challenge experiment was carried out using JHT mice. JHT mice lacking a functioning gene for antibody heavy chain production are a model devoid of mature B lymphocytes. Groups (n = 10) of adult JHT mice were immunized twice with recombinant HSV-GS19 (2.5 × 10^5^ PFU/animal) or were mock-immunized, respectively. All mice were then challenged with a lethal dose of the influenza virus strain HK14, and their survival and weights were recorded over a 30-day observation period. Vaccination with RCCV HSV-GS19 nearly completely protected the mice against the lethal cross-group challenge, suggesting that the vaccination induces a broad HA-directed effector T-cell response (Figure 5c, left graph). Unlike in the above-described experiments that employed immunocompetent mice, immunized JHT mice lost weight after the challenge (center and right panels). Taken at face value, this finding may suggest that the HA-directed antibody response trumps the effector T-cell response in immunocompetent mice.

## 4. Discussion

Activated RCCVs expressing a full-length influenza A virus HA arguably induced broader and more effective protective responses against influenza A viruses in mice than any other monovalent HA-directed vaccine that has been investigated to date [1,2,3,4,5,6,7,8,9,10,11,12,13,14,15,16,17,18,19,20,21,22,23,24,25,26,27]. Highly efficacious cross-group protection against lethality (100% survival) was achieved using RCCVs expressing either an H1 or an H3 HA. No significant illness or weight loss was observed after the lethal challenge. Protective effects even extended to influenza type B. Broad heterologous protection also resulted from vaccination with an RCCV expressing an influenza virus NA. We note that a recent study evaluated the vaccine potential of an HSV-2-derived disabled infectious single-cycle (DISC) virus expressing an influenza virus subtype H1 HA under the control of a CMV IE promoter [45]. While protecting effectively against a challenge by the homologous influenza virus strain, this vaccine was incapable of affording detectable protection against a heterologous H1N1 virus strain or an H3N2 virus strain. As our own unpublished studies confirmed, DISC viruses expressing an influenza virus HA do not appear to induce elevated levels of influenza virus-neutralizing antibodies, and the latter study provided evidence that protective effects may be mediated by antibody-dependent cellular cytotoxicity (ADCC). Explanations for the dramatically different abilities of the latter DISC vaccine and our RCCV-based vaccines to induce broadly protective immune responses may be found in the different properties of the vaccines and/or vaccination procedures. The DISC vaccine was derived from an attenuated HSV-2 strain, whereas our RCCV vaccines are based on a highly virulent HSV-1 strain. The DISC vaccine produces non-infectious progeny, whereas RCCV vaccines expressing all HSV proteins are expected to give rise to infectious (albeit non-replicating) progeny. Furthermore, RCCV vaccines require activation, which includes a heat treatment of the vaccine virus-infected host cells. Heat treatment appears to boost the protective response if it is coordinated with the replication of the vaccine virus (our unpublished observations). 

Passive immunization experiments suggested that the observed protective effects of RCCVs expressing an influenza virus HA were brought about at least in part by antibody responses directed to the HA antigens presented. This was further supported by the detection of robust serum titers of neutralizing antibodies in HA-immunized animals. Our experiments evaluated the protective effects of vaccination with three different doses of RCCV HSV-GS19 against a lethal challenge by a heterologous H1N1 influenza virus strain (Figure 3). In the group vaccinated with the lowest dose (1 × 10^4^ PFU/animal), 40% of the animals survived, and the remaining animals succumbed to the challenge (panel g). All animals lost weight after being challenged prior to the recovery of the survivors. In groups vaccinated with the highest dose (2.5 × 10^5^ PFU/animal), all animals survived the challenges, and all gained weight normally (panels b, c and d). An unexpected pattern was observed with animals immunized with the intermediate vaccine dose (5 × 10^4^ PFU/animal): a majority of the animals in the groups survived the challenge and gained weight normally (90% of the group in panel e and 70% in panel f). The remaining animals lost weight rapidly and died. None of the animals exhibited a partial vaccination effect (i.e., transient weight loss). Analogous observations were made in the experiments in which animals vaccinated with an influenza A virus HA were challenged with an influenza B virus (Figure 4d,e). Perhaps an early process, such as the activation of broad reactivity-generating immune cells, e.g., B cells producing broadly neutralizing antibodies, and a subsequent process, such as the elaboration of protective levels of these immune cells, i.e., their proliferation and migration, are independently dependent on vaccine dose. If this were the case, it would be conceivable that in some animals of a group vaccinated with the intermediate vaccine dose, the latter immune cells were not activated, causing the animals to succumb to the heterologous challenge. In the other animals, these immune cells may have been activated and produced at a sufficiently elevated level to fully protect them against the challenge. In a group of animals immunized with the low vaccine dose, a greater number of animals may not have had activated immune cells and were killed by the challenge. In the remaining animals of the group, the immune cells may have been activated but were only available in the tissue of interest at a relatively low level that, upon challenge, protected the animals against lethality but not illness. Even after immunization with the high vaccine dose, a fraction of the animals may not have expressed activated immune cells capable of countering an influenza B virus challenge. The other animals that may have expressed such immune cells produced them at a high level that fully protected them against the influenza B virus challenge. 

While our findings suggest that influenza virus-neutralizing antibodies contribute importantly to the observed protective effects, this neutralizing antibody response may be supported by other facets of the immune response. This includes the potential role of ADCC, which we have not investigated. Viruses are well-known to induce powerful T-cell responses. The use of viral vectors to increase cellular responses to vaccines has been a field of intensive investigation [46,47]. Perhaps not surprisingly, we were able to obtain evidence suggesting that vaccination with an RCCV expressing an influenza A virus HA induced an effector T-cell response that was protective across influenza A virus groups. We note that in a recent study, vaccination with a recombinant CMV expressing a full-length influenza virus HA was found to protect mice against a homologous lethal challenge [48]. Somewhat unexpectedly, the authors found that this protection was largely antibody-mediated and that the T-cell response did not significantly contribute. 

Several unique features distinguish the RCCV vaccination approach. RCCVs are conditionally replicating recombinants derived from a virulent HSV-1 strain. They are designed to not replicate unless activated. Activation (heat treatment to the inoculation site in the presence of an AP in the case of the first-generation RCCVs discussed herein) triggered highly efficient but transient replication in the inoculation region. Administration of the RCCVs was to the epidermis, which is where the normal host cells of HSV-1, primarily keratinocytes, abound. The RCCVs expressed an unmodified, full-length influenza virus HA or NA. Hence, the immune response was not intentionally directed to a particular domain of the antigens, such as the stem domain of HA, or to particular natural or optimized conserved epitopes. To the extent that we can infer from a side-by-side comparison of RCCV vaccines HSV-GS19 and HSV-GS25, forcing the immune system to respond to the stem domain rather than the entire HA protein does not appear to result in a stronger protective response against a heterologous influenza virus.

Our data reveal that an effective protective response against an influenza virus could not be obtained without the activation of RCCV replication. We previously made analogous observations in HSV-1 challenge experiments [39]. We can only speculate as to why this is the case. Perhaps, as we had hypothesized previously [49], ample production of progeny virus and unassembled viral proteins liberated by host cell lysis strongly stimulates the host’s inflammatory response, resulting in a particularly robust and balanced immune response. Induced replication of RCCV DNA amplifies the production of viral proteins as well as that of a virus-encoded influenza virus surface antigen, as reported previously [38,39]. This may result in an enhanced display or presentation of such surface antigen on the infected host cells. In addition, large quantities of the influenza virus antigen, in oligomerized or monomeric form, and, possibly, of progeny RCCVs displaying the antigen may be released subsequently when the host cells are lysed. Perhaps, in the case of surface proteins such as HA or NA, glycosylation may not keep pace with the high rate of their induced expression and deployment to the cell membrane or, possibly, the progeny virus envelope. A recent study demonstrated that an unglycosylated HA induced a broader protective response than the corresponding fully glycosylated HA [50].

Our studies in mice suggest that RCCVs expressing an influenza virus HA possess key attributes that are sought in a universal or pandemic influenza vaccine, i.e., the ability to induce a broadly protective immune response, the ability to largely prevent any disease manifestation and the expected ability to reduce pathogen spread by vaccinated and subsequently infected subjects as well as the inability of the vaccine to propagate in an uncontrolled fashion in immunocompromised subjects (a danger inherent in attenuated vaccines). We expect future RCCV-based influenza vaccines for use in humans to express at least one HA from an influenza A strain and one HA from an influenza B strain. An HA from an influenza B strain may need to be presented owing to the apparent inability of type A HAs to generate fully protective responses against influenza B viruses. Robustness may be enhanced by presenting an additional influenza A virus HA. Multiple HAs may be expressed from a single RCCV or from different RCCVs. Our limited studies suggest that RCCV-delivered influenza virus NAs may also be broadly protective. Hence, an HA-directed RCCV vaccine may also be strengthened by the co-presentation of an NA. Obviously, our findings will need to be validated in human clinical trials, and the durability of immune responses to RCCV vaccines will have to be assessed in human subjects. That natural infection and vaccination by live attenuated influenza virus vaccines induce considerably longer-lasting immune responses than conventional subunit or inactivated virus vaccines nurtures the expectation that immune responses to activated (replicating) RCCVs may be long-lived [2,51,52].

Data reported herein and in ref. [39] suggest the possibility that our RCCV-based influenza vaccines may double as HSV-1 vaccines. Because HSV-1 and HSV-2 are highly related, the vaccines might also show some effectiveness against HSV-2. It is noted that RCCVs may also be derived from a virulent HSV-2 strain. Children younger than five years, and especially children under the age of two, are considered to have an increased risk for complications from influenza virus infection (https://www.cdc.gov/flu/highrisk/children.htm, accessed on 5 April 2024). Hence, vaccination against influenza arguably should occur as early as possible. The prevalence of HSV-1 infection in 5-year-old children has been estimated to be around 10% in the Americas and increases rapidly with age [28]. Vaccination against HSV-1 at an early age would clearly be desirable. Although RCCV-based vaccines may not produce sterilizing immunity against HSV-1, they could be expected to reduce disease manifestations and discomfort as well as viral spread. 

As mentioned above, it may be desirable to vaccinate the very young with a broad anti-influenza/anti-HSV-1 vaccine. Our first-generation RCCVs employ a dual-responsive gene switch for stringently controlling viral replication. Activation requires both heat treatment and the administration of a drug. There may be a reluctance to the administration of a drug to young children for a reason that is only indirectly related to therapy, even though it might be given topically and/or at a subclinical concentration. The purpose of the drug co-control was to increase vaccine safety, particularly to prevent systemic replication and replication in nerve cells. We have recently developed second-generation, heat-activated RCCVs, in which one replication-essential viral gene is controlled by the HSP70B promoter and another by a keratin gene promoter. These RCCVs replicate efficiently in skin cells but not nerve cells (and most other cell types), obviating the need for a drug-based co-control. In the animal studies presented herein, as well as previously [39], RCCV vaccines were administered epidermally, and the second-generation RCCV vaccines were also designed to be administered by this route. Epidermal vaccination of human subjects by skin scarification may be disfavored, and epidermal injection of a vaccine requires a certain level of skill as well as may be painful. We surmise that an RCCV-based vaccine may be best delivered by means of a microneedle patch. Microneedle patches containing live enveloped viruses have been developed before and have been tested successfully in vaccination experiments [53,54]. Heat treatment would be administered subsequent to microneedle delivery of the RCCV vaccine. We demonstrated previously that a 15 min application of a simple heating pad to the forearm of a human subject results in a strong local activation in all skin layers of the HSP70B promoter that drives RCCV replication [55]. The heat was produced by the crystallization of a supercooled solution of a readily available, nontoxic salt. It is noted that a vaccination procedure involving the sequential application of a vaccine-containing microneedle patch and a heating pad would not depend on the availability of medically trained personnel and could be practiced anywhere (possibly without requiring a cold chain).

Numerous previous studies addressed the question of whether pre-existing immunity to HSV impairs the efficacy of HSV-based vaccines or oncolytic HSVs [56,57,58,59,60,61,62,63]. A majority of these studies reported no more than minor effects. Only two studies that investigated untypical experimental scenarios described substantial negative effects [61,62]. All studies agreed that pre-existing immunity should not be an obstacle to vaccine uses of HSV. We previously found that a single epidermal immunization of mice with an RCCV protected about 70% of the animals against a lethal HSV-1 challenge [39]. A second immunization 3 wk after the first immunization resulted in complete protection, indicating that the second immunization was effective in the face of pre-existing immunity. A recent study employing an alpha-herpesvirus recombinant expressing an influenza virus HA concluded that pre-existing immunity to HSV did not decrease the ability of the recombinant to induce a protective immune response directed against the HA [45]. Hence, pre-existing immunity to HSV is not expected to significantly affect the efficacy of RCCV-based influenza vaccines. Epidermal administration may be particularly advantageous in this regard as it brings RCCVs into immediate contact with their normal host cells (i.e., keratinocytes), limiting premature encounters of the recombinants with components of the immune system. It is also noted that pre-existing immunity should be of lesser concern in the case of vaccination of young children. 

Broadly protective immune responses against influenza viruses were not only induced by RCCVs expressing an HA but also by an RCCV expressing an NA. These findings suggest that the quality of the immune response elicited by the RCCV-based vaccines was not critically dependent on the nature of the influenza virus surface antigen that they caused to be presented. This raises the possibility that the RCCV approach may also be adapted to vaccinate against other RNA viruses of concern, such as severe acute respiratory syndrome coronavirus 2, respiratory syncytial virus or even human immunodeficiency virus.

## Figures and Tables

**Table 1:** RCCVs (all derived from HSV-GS3) employed in the present study.

RCCV	Influenza Virus Antigen Expressed
HSV-GS3	None
HSV-GS19	Full-length HA of strain A/California/07/2009(H1N1) (CA09)
HSV-GS21	Full-length NA of strain A/California/07/2009(H1N1) (CA09)
HSV-GS25	HA stem region miniprotein (H1HA10-Foldon A) derived from strain A/Puerto Rico/8/1934(H1N1) (PR34)
HSV-GS26	Full-length HA of strain A/Hong Kong/4801/2014(H3N2) (HK14)
HSV-GS27	Full-length HA of strain A/Perth/16/2009(H3N2)

## Data Availability

Data presented in this study are available upon reasonable request from the corresponding author.
